# Multimodal Role of Amino Acids in Microbial Control and Drug Development

**DOI:** 10.3390/antibiotics9060330

**Published:** 2020-06-17

**Authors:** Muhammad Idrees, Afzal R. Mohammad, Nazira Karodia, Ayesha Rahman

**Affiliations:** 1 Faculty of Science and Technology, University of Wolverhampton, Wolverhampton WV1 1LY, UK; M.Idrees@wlv.ac.uk (M.I.); nazira.karodia@wlv.ac.uk (N.K.); 2 Aston Pharmacy School, Aston University, Birmingham B4 7ET, UK; a.u.r.mohammed@aston.ac.uk

**Keywords:** amino acids, antimicrobial resistance, quorum sensing, microbial biofilm, solubility, excipients, adjuvants

## Abstract

Amino acids are ubiquitous vital biomolecules found in all kinds of living organisms including those in the microbial world. They are utilised as nutrients and control many biological functions in microorganisms such as cell division, cell wall formation, cell growth and metabolism, intermicrobial communication (quorum sensing), and microbial-host interactions. Amino acids in the form of enzymes also play a key role in enabling microbes to resist antimicrobial drugs. Antimicrobial resistance (AMR) and microbial biofilms are posing a great threat to the world’s human and animal population and are of prime concern to scientists and medical professionals. Although amino acids play an important role in the development of microbial resistance, they also offer a solution to the very same problem i.e., amino acids have been used to develop antimicrobial peptides as they are highly effective and less prone to microbial resistance. Other important applications of amino acids include their role as anti-biofilm agents, drug excipients, drug solubility enhancers, and drug adjuvants. This review aims to explore the emerging paradigm of amino acids as potential therapeutic moieties.

## 1. Introduction

Amino acids are important biomolecules and the very basic constituents or building blocks of proteins [1]. They have at least one amino group, one carboxyl group, and a side chain in their structures [2,3]. There are 20 different amino acids, represented by a general formula H2N-CαH(R)-CO2H (where -R is a side chain with an exception of glycine, in which the side chain -R has been replaced by -H), which are responsible for protein formation in all living organisms [4,5].

**Figure 1:**
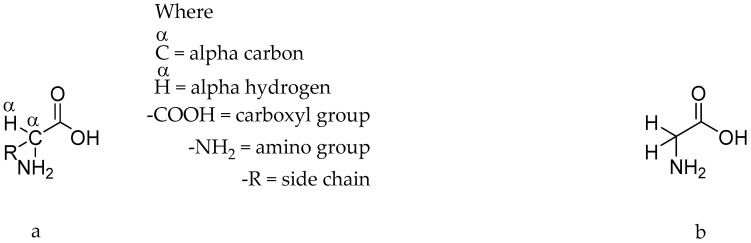
(**a**) depicts a general chemical structure of an amino acid; (**b**) is the structure of amino acid glycine.

Several amino acids (in different proportions) are joined together by amide bonding (also known as peptide bonding) in such a way that the carboxyl group of one amino acid links to the amino group of another amino acid with the loss of a water molecule (per each peptide bond) to form protein polymers [6]. Figure 1a,b illustrates a general chemical structure for 19 of the 20 amino acids and glycine, respectively.

Besides the most common 20 proteogenic amino acids, there are hundreds of other non-protein amino acids with varied biological functions, found in all kinds of living systems including humans, plants (abundantly found in legumes and seed), bacteria, and fungi [7,8,9]. Unlike proteogenic amino acids, non-proteogenic amino acids are not naturally encoded with a genetic code and therefore, are also referred to as non-coded amino acids [10]. Non-proteogenic amino acids are secondary metabolites, produced from natural proteogenic amino acids and are usually toxic in nature [11]. Hypoglycine (also written as hypoglycin), canavanine, and mimosine are examples of non-proteogenic amino acids [9].

Non-proteogenic amino acids are able to pose themselves as if they have certain properties such as chemical structure, size, shape, and charge, etc., similar to those exhibited by normal proteogenic amino acids [12]. By mimicking one or more of these properties, non-proteogenic amino acids are mistaken for normal amino acids and during protein biosynthesis, they are incorporated into different proteins [13]. This incorporation of non-proteogenic amino acids results in the formation of unnatural proteins with improper functionalities and hence causes undesired consequences [9]. Azetidine-2-carboxilic acid is a non-proteogenic amino acid (found in sugar beets), due to structural similarities, behaves the same way as alanine and proline [14]. Azetidine-2-carboxilic acid, if consumed during pregnancy, is believed to be the cause of multiple sclerosis in newborns [13].

Amino acids play an important role in microbial metabolism and in facilitating microbial growth, microbial biofilm formation, and its dispersal [15]. Based on the source of amino acids and nature of the side chain, amino acids can be categorised as essential and non-essential amino acids, acidic, basic and neutral amino acids, polar and nonpolar amino acids, hydrophilic and hydrophobic amino acids [16].

Essential amino acids are those amino acids that cannot be synthesised by living organisms and must be supplemented from dietary means [17]. Essential amino acids such as isoleucine, leucine, lysine, methionine, phenylalanine, taurine, threonine, tryptophan, valine, arginine, glycine, and histidine are the common ones found in almost all living organisms in varied proportions [18]. Non-essential amino acids are biosynthesised inside the cells of living organisms and are rarely required to be supplemented or obtained from dietary sources [17]. Non-essential amino acids include alanine, aspartate, asparagine, arginine, cysteine, glutamate, glutamine, glycine, proline, serine, and tyrosine [19].

This review is an effort to highlight different physicochemical properties of amino acids, their microbial biosynthesis, their role in intermicrobial interaction as well as microbe-host interactions, and more importantly, the role played by amino acids in the fight against antimicrobial resistance (AMR) as potential antimicrobial agents, antibiofilm agents, drug adjuvants and drug excipients.

### Types of Amino Acids and Chirality

Amino acids can be classed as acidic, basic, or neutral amino acids on the basis of their side chain. An acidic amino acid possesses an acidic functional group in its side chain and a basic amino acid has a basic functional group attached to its side chain, while a neutral amino acid has neither an acidic nor a basic functional group attached to its side chain [4].

**Figure 2:**
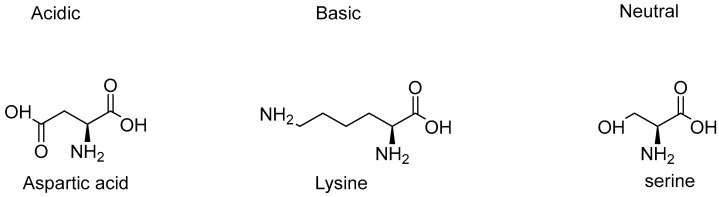
Chemical structures of acidic, basic and neutral amino acids.

Examples of acidic amino acids are aspartic acid and glutamic acid, basic amino acids include lysine, arginine, and histidine, while serine threonine and tyrosine are examples of neutral amino acids [20,21]. Depiction of chemical structures, each from acidic, basic, and neutral amino acids have been given in Figure 2.

The most common 20 proteogenic amino acids (with the exception of glycine) exhibit chirality as the central carbon in their molecules is attached to four different groups, enabling each of these amino acids to exist as two mirror images that are non-superimposable which are called enantiomers [22]. Glycine being the only achiral amino acid as has two hydrogens attached to the central carbon i.e., the side chain -R = H [4].

**Figure 3:**
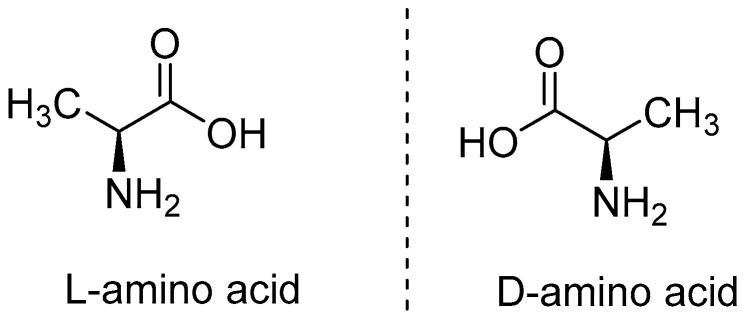
Two enantiomeric forms, l- and d- of an amino acid.

Both enantiomers (isomers) of an amino acid have similar properties but differ in their interaction with plane-polarised light i.e., one enantiomeric form rotates the light towards the right side and is called dextro- (right handed) rotatory, abbreviated as d-, whilst the other form rotates the light towards the left side and is therefore referred to as laevo- (left handed) rotatory, abbreviated as l- [23]. Figure 3 is a general representation of d- and l- form of a typical amino acid.

With the exception of glycine, which is an achiral amino acid, all other 19 amino acids have a center of chirality and exist in two enantiomeric forms i.e., l-amino acids and d-amino acids [24].

**Figure 4:**
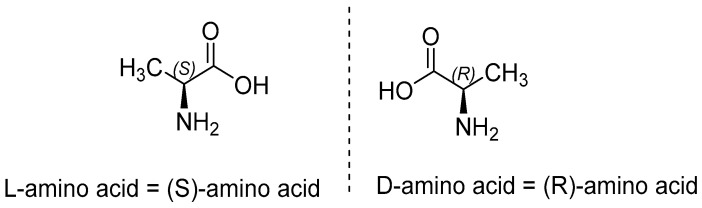
(S) and (R)-enantiomers of an amino acid, representing its respective l and d-enantiomers.

In chemistry, d-enantiomer of an amino acid is also referred to as its (R)-enantiomer and l-enantiomer as its S-enantiomer, where “R” and “S” both come from Latin words rectus and sinister (right-handed and left-handed respectively). This type of nomenclature follows Cahn–Ingold–Prelog (CIP) convention, that assigns priorities (based on atomic numbers or atomic mass) to different constituents attached to the asymmetric central carbon [25]. According to R, S system, all l-amino acids are (S)-amino acids with the exception of l-cysteine i.e., l-cysteine is not an (S)-cysteine but is an (R)-cysteine instead [26]. Figure 4 depicts the (S) and (R)-enantiomeric structures for the respective l and d-enantiomers an amino acid.

Chirality of amino acids is of significant importance when it comes to peptide drug designing and synthetic peptide chemistry [27]. It also plays a significant role in determining if a particular amino acid will participate in protein formation or it will serve to regulate other cellular functions. For instance, l-amino acids are proteogenic and will form proteins as well as participate in microbial cell wall formation, whereas d-amino acids are non-proteogenic and regulate different functions such as microbial cell wall formation and dispersal of microbial biofilm [28,29]. The chiral behaviour of amino acids is also responsible for stereoselective catabolism of amino acids i.e., D-amino acids will be catabolised only by d-amino acid oxidase (DAAOs) enzymes and l-amino acids will have their corresponding l-amino acid oxidases (LAAOs) for their catabolism [30].

Chirality of amino acids also stereo-select the antimicrobial properties of LAAOs and DAAOs i.e., LAAOs will exhibit antibacterial activity only in the presence of corresponding substrate (l-amino acids) and DAAOs will exhibit antibacterial activity in the presence of d-amino acids [31,32]. However, the antibacterial mechanism of both DAAOs and LAAOs remains the same i.e., the generation of hydrogen peroxide (H_2_O_2_) during the oxidative deamination of stereo-selected amino acids [33].

## 2. Microbial Biosynthesis of Amino Acids

Microbial biosynthesis of amino acids requires carbon that comes from either the citric acid cycle, glycolytic pathway or pentose phosphate pathway, and nitrogen that comes from ammonia (microbes convert nitrogen gas into ammonia) [34]. Microorganisms are generally capable of producing all the required proteogenic amino acids, however, the mechanism of amino acid biosynthesis is different in different microorganisms [35,36].

Different organic intermediates and precursors undergo biochemical reactions and form different amino acids [37]. Some precursors such as oxaloacetate from the citric acid cycle, α-ketoglutarate from Krebs cycle, and 3-phosphoglycerate from Calvin cycle are converted into amino acids that act as intermediates in the biosynthesis of other amino acids [34,38,39,40]. Table 1 enlists different precursors for the respective intermediates and amino acids.

### 2.1. Fungal Biosynthesis of Amino Acids

**Figure 5:**
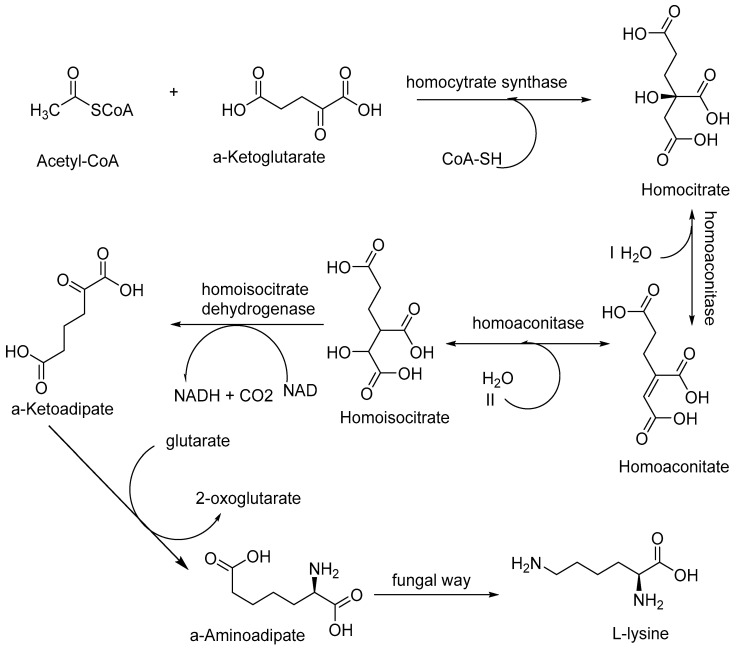
Schematic representation of l-lysine biosynthesis via α-aminoadipate pathway. It illustrates that precursors acetyl-CoA and α-ketoglutarate in the presence of homocitrate synthase form homocitrate (a tricarboxylic acid). Homoaconitase enzyme dehydrates homocitrate to form homoaconitate (homoaconitatic acid) that leads to the formation of homoisocitrate in the presence of homoaconitase and water. Homoisocitrate dehydrogenase converts homoisocitrate into α-ketoadipate. NAD is converted into NADH and CO_2_ is generated during this step. Now, glutamate reacts with α-ketoadipate and forms α-amino adipate which converts into l-lysine. 2-oxoglutatarte (de-amination of glutamate) is also generated in the process. Concept adopted (and modified) from Fazius, F., 2012, Jastrzebowska, K., 2015 and Miyazaki, J., 2001 [41,42,43].

Fungi synthesise all the amino acids required for protein synthesis and other metabolic activities using different enzymes. The mechanism of biosynthesis of certain amino acids (that cannot be synthesised by mammals) has been reported to be similar in fungi and bacteria with the exception of biosynthesis of l-lysine through α-aminoadipate pathway which is only found in fungi. Biosynthesis of lysine is catalysed by enzymes such as homocitrate synthase, homoaconitase, homoisocitrate dehydrogenase, α-aminoadipate aminotransferase, α-aminoadipate reductase, saccharopine reductase, and saccharopine dehydrogenase [41,42,43] as shown in Figure 5.

Despite being able to biosynthesise amino acids, fungi have also evolved certain mechanisms such as nitrogen catabolic repression (NCR), transceptor-mediated amino acid sensing, Ssy1-Ptr3-Ssy5 (SPS) and target of rapamycin (TOR) pathway to monitor and use the amino acids present in the environment [44]. SPS is used to sense and uptake exogenous amino acids and TOR serves as a sensory system to monitor intracellular amino acids [45].

### 2.2. Bacterial Biosynthesis of Amino Acids

**Figure 6:**
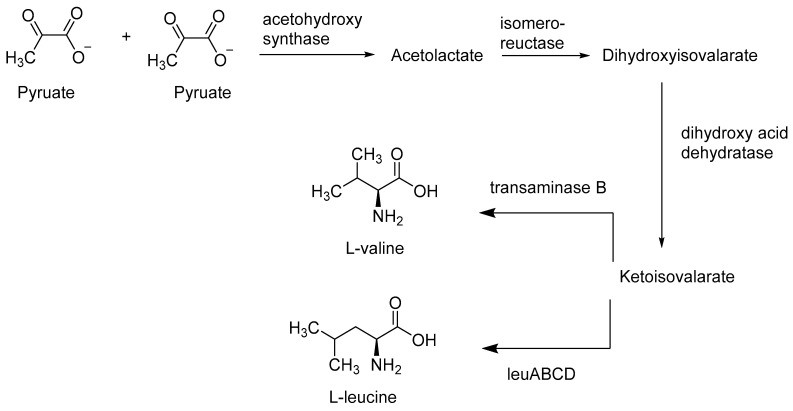
Schematic representation of branched chain amino acids in bacteria. It shows how two pyruvate molecules (product of glycolysis) in the presence of acetohydroxy acid synthase react to form acetolactate. Isomero-reductase enzyme converts acetolactate into an intermediate called dihydroxyisovalerate which, in the presence of dihydroxy acid dehydratase enzyme, dehydrates and produces the final intermediate, ketoisovalerate. Enzyme transaminase B converts ketoisovalerate into l-valine, while enzyme leu BCD converts it into l-leucine. Concept adopted from Amorim Franco, T.M., 2017 [38].

Bacteria such as *Escherichia coli* can biosynthesise all the required 20 amino acids (proteogenic amino acids), including the essential ones, while others such as *Lactobacillus plantarum* need to acquire them from their external environment [38,46,47,48]. Several enzymes participate to catalyse the formation of each amino acid e.g., bacterial biosynthesis of branched-chain amino acids (leucine, isoleucine, and valine) for instance, is catalysed by at least eight different enzymes i.e., l-threonine dehydratase, acetolactate synthase, keto acid isomeroreductase, dihydroxy acid dehydratase, isopropylmalate synthase, isopropylmalate isomerase, isopropylmalate dehydrogenase, and branched-chain aminotransferase [38,49]. Figure 6 illustrates various steps involved in bacterial biosynthesis of branched amino acids.

Due to a variety of industrial applications (pharmaceuticals, cosmetics, animal feeds, etc.) of amino acids, microbial biosynthesis of amino acids has been adopted as a means of large-scale production by the process of fermentation [50]. The fermentation process involves the growth of microorganisms on less expensive growth media on the industrial level to produce tons of high-quality amino acids each year e.g., industrial production of l-glutamic acid and l-lysine [51,52,53].

## 3. Antimicrobial Resistance (AMR) and Role of Amino Acids

Antimicrobial resistance (AMR) is not only taking its toll on the world’s human and animal population, but is also causing serious financial losses to the global economy [54]. According to a report published by the World Health Organisation (WHO) on AMR related worldwide mortalities, at least 700,000 people die each year because of AMR. This number expected to escalate to 10 million per year by the year 2050 [55]. To avert serious pandemics such as the situation due to AMR, the world needs novel therapeutic strategies such as the development of novel antimicrobial drugs as well as repurposing the existing ones, including the drugs that were previously abandoned for different reasons.

Microorganisms have developed different strategies to resist antimicrobial drugs, either by reducing their efficacy or by rendering the drugs fully inactive. Different mechanisms through which microbes develop and spread resistance against antimicrobials include efflux pumps, modification of drugs, alteration of the target sites, decrease in cellular permeability to reduce drug penetration, enzymatic degradation of antimicrobial compounds, and biofilm formation [56].

Microbial cellular envelopes have protein-made designated transport channels called porins [57]. These porins act in the same way as efflux pumps that expel antimicrobial drugs out of the cell, thereby reducing the drug concentration to avoid cytocidal effects [58]. The lipophilic outer membrane in Gram-negative bacteria limits the passage of hydrophilic drugs through porins (protein channels meant for the passage of substances) to a level where the drug concentration is no longer bactericidal [59].

Amino acids (in the form of enzymes) also contribute to antimicrobial resistance in different ways i.e., drug modification, and inactivation via enzymatic degradation e.g., phosphoethanolamine transferase, hydrolases and redox enzymes [60]. Enzymes such as amidases and acyl transferases degrade the antimicrobial drugs (β-lactams and macrolides) while others such as epoxidases, macrolide esterases, etc., modify the antimicrobial drugs (rifamycin, aminoglycoside, etc.) and help the microbes to resist the antimicrobial effect of the drugs [61].

Alongside other strategies (such as developing novel drugs and repurposing the existing ones), amino acids can also be important drug candidates in the fight against antimicrobial resistance, both as antimicrobial as well as antibiofilm agents [62]. Amino acids have been used to produce antimicrobial peptides that are more efficient as antimicrobial and antibiofilm agents and less prone to resistance [63,64]. Amino acids have also been used to increase the efficacy (in vitro) of existing drugs i.e., the efficacy of antimicrobial drug trimethoprim has been enhanced by amino acids-based trimethoprim salt formation [65].

Other applications of amino acids in the fight against AMR, include their use as antibiofilm agents, solubility enhancers for existing drugs and their physical combination with antimicrobial drugs to enhance their efficacy by additive effect or synergism [62,66,67,68]. Different roles and applications of amino acids in microbial control and tackling AMR, have been discussed in the following sections.

## 4. Role of Amino Acids in Microbial Infections and Quorum Sensing

In any particular microbial-host environment, microbes and their host communicate at the site of infection [69,70]. D-amino acids, in particular, which facilitate communication amongst the microbial communities, play a role in microbial-host interaction as well as affect the host immune system by functioning as host defense peptides [71,72,73].

Trillions (10–100 trillions) of microorganism including archaea, bacteria, fungi, viruses, and protozoans inhabit the human body (both, externally and internally), living in a symbiotic environment collectively known by the term “microbiota” whereas the total number of microbial genes in a human microbiota are referred to as the human microbiome [74,75,76].

The human microbiome, particularly hosted by the human gut (also known as a virtual organ within an organ due to its collective metabolic activity), plays an important role in homeostasis (a balanced, stable physical and chemical environment) and immune system, and any imbalance in the microbiome can have its consequences in the form of pathogenic and other diseases [77,78].

The symbiotic relationship is beneficial for human gut microbiota and the host itself, where the microbes (bacteria in particular) break down the available proteins in the host environment and utilise the resultant amino acids, while their human host is benefited from the amino acids secreted by the microbes [79]. These microbial amino acids can, sometimes, if secreted in excess, can disturb the normal human microbiota (a condition also known as dysbiosis), leading to the production of short-chain fatty acids (SCFA) causing liver problems, obesity and diabetes [80].

Microorganisms exchange cell to cell information and communicate to one another through an intercellular microbial system of communication, known as quorum sensing (QS) [81]. Microbial quorum sensing is activated by certain extracellular chemical signals, called autoinducers [82,83]. Autoinducers that activate the quorum sensing in Gram-positive bacteria are peptides [84]. The quorum sensing peptides (QSP) are composed of different amino acids such as serine, tyrosine, tryptophan, isoleucine, leucine, cysteine, threonine, glycine, glutamic acid, phenyl alanine, proline, and valine [85]. Quorum sensing plays a key role in controlling different microbial properties such as microbial biofilm formation, antimicrobial resistance and microbial infections [86].

The initiation of microbial infection leads to the activation of host immune system which prompts a hypercatabolic response from the host and thus results in an increased consumption of host amino acids and other nutrients such as vitamins, fatty acids, etc. [87]. Microbial infections cause amino acid depletion and a loss of host body proteins, hence it is important to provide the infected host with extra dietary proteins and amino acids (20 to 25% extra as compared to normal intake) during any microbial infection-mediated hypercatabolic phase and the recovery phase, to stop any further depletion of proteins [88].

Amino acids regulate different biological events in microbes such as spore germination, microbial growth, and remodeling of the microbial cell wall [28]. Microbial infections are a direct consequence of microbial growth as microbes utilise amino acids as a source of nutrients and energy for their survival [89,90].

Microorganisms such as bacteria and fungi, when faced with adverse environmental conditions (either living freely or on/inside a host) such as lack of essential nutrients, lack of moisture or even when under the influence of toxic chemicals and high temperatures, produce certain types of highly infectious cells, called spores which can survive the aforementioned extreme conditions [91,92]. Spores germinate into active cells after sensing certain biomolecules, known as germinants (which are amino acids and sugar molecules), and cause pathogenic illnesses [93]. Amino Acids also play an important role in the development of microbial appendages (such as pili in bacteria and hyphae in fungi), that are used for nutrients uptake, locomotion and more importantly for adhesion to the host surface, thereby help in developing and spreading microbial diseases [28,94,95,96].

Despite their role in the development and spread of microbial infections, certain peptides and amino acids, d-amino acids in particular, have been reported to exhibit broad-spectrum antimicrobial, anti-quorum sensing as well as antibiofilm activity. They are less likely to be resisted by microbes, hence offer an advantageous alternative to traditional antimicrobial drugs [97,98,99,100]. The aforementioned different roles of amino acids have been discussed in detail, in the forthcoming sections.

## 5. Role of Amino Acids in Bacterial Cell Wall Formation and Controlling Planktonic Bacteria

Amino acids perform different functions in planktonic bacteria, ranging from exhibiting protective roles to being used as nutritional substrates. Bacterial peptidoglycan (PG), an important functionary in the biosynthesis of the bacterial cell wall, has amino acid constituents in it [101]. PG is a polymer that gives strength to the bacterial cell wall and has sugar molecules alongside its amino acid residues [102]. PG relies on penicillin-binding proteins (PBP) for its synthesis, while its strength and elasticity are dependent on amino acids that regulate the function of PBP [103].

The synthesis of peptidoglycan takes place in the cytoplasm in three stages i.e., i) synthesis of nucleotide precursors namely UDP-*N*-acetylglucosamine and UDP-*N*-acetylmuramyl pentapeptide, ii) assembly of precursors into monomer subunits and iii) polymerisation of monomers in the presence of glycosyltransferases [104]. The precursor nucleotide UDP-*N*-acetylglucosamine is synthesised from fructose-6-phosphate in the presence of enzyme glucoamylase (Glm), the precursor UDP-*N*-acetylglucosamine is converted into nucleotide UDP-*N*-acetylmuramyl pentapeptide by MurA, MurB, MurC, MurD, MurE and MurF enzymes [105,106]. A typical pentapeptide has -(l-alanine-d-glutamine-l-lysine-d-alanine-d-alanine)- sequence of amino acids [107].

**Figure 7:**
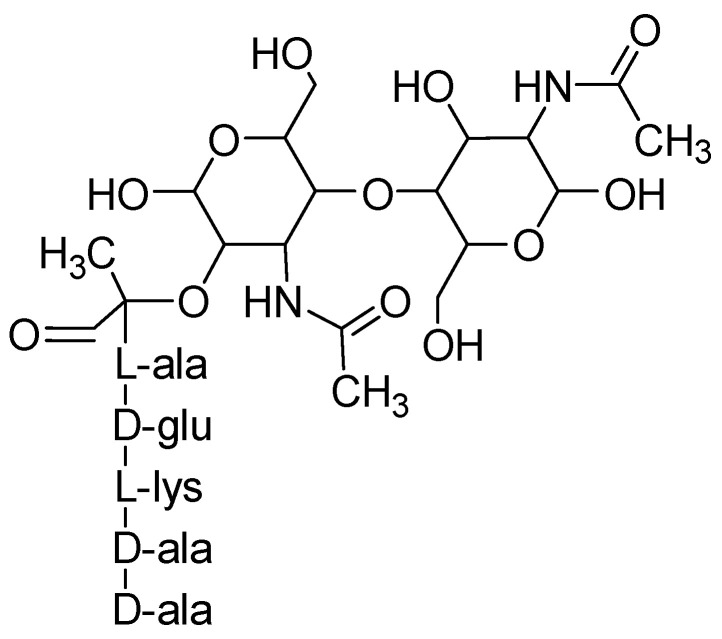
Disaccharide pentapeptide monomeric unit of peptidoglycan.

The precursor nucleotides are then bonded to undecaprenyl phosphate and form the monomeric lipid-anchored disaccharide pentapeptide units (also known as lipid II) which are cross-linked and polymerised by enzymes glycosyltransferases and penicillin-binding proteins (PBPs) to form peptidoglycan layers [104,108,109]. Figure 7 is a depiction of disaccharide pentapeptide monomer of peptidoglycan.

**Figure 8:**
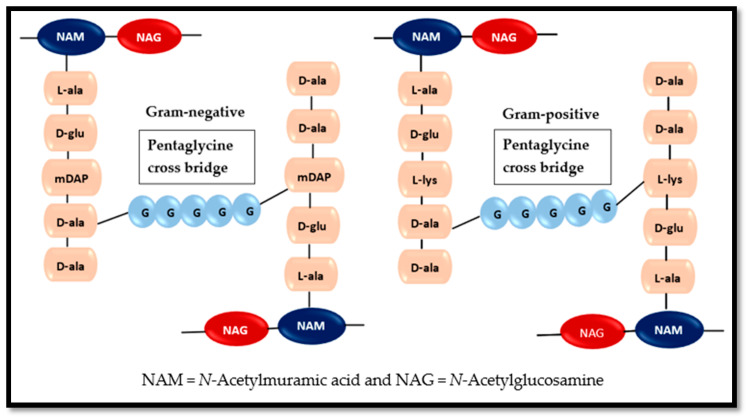
Pentaglycine cross-linkages in Gram-negative and Gram-positive bacterial peptidoglycan. Disaccharide pentapeptide monomer in Gram-negative bacterial peptidoglycan possesses meso-diaminopimelic acid (mDAP), whereas Gram-positive bacterial peptidoglycan has l-lysine instead. Basic concept (with adaptations) has been drawn from Irazoki, Hernandez, and Cava, 2019 [111]. Figure 8 has been drawn using Microsoft Word.

The basic two-dimensional structure of peptidoglycan mesh remains the same in both Gram-positive and Gram-negative bacteria i.e., glycan strands are oriented in circumferential order and crossly linked by peptide side chains [110]. Figure 8 is a general depiction of pentaglycine cross-linkages in Gram-negative and Gram-positive bacterial peptidoglycan.

Gram-positive bacteria possess multilayers (10 or more) of peptidoglycan and is about 50% of the cell wall weight, whereas the peptidoglycan in Gram-negative bacteria is 1–2 layered, constituting 10–20% of the cell envelop weight [112,113].

**Figure 9:**
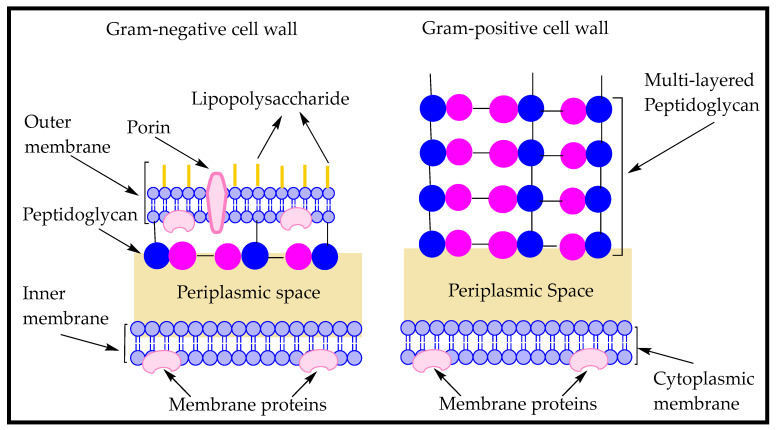
Gram-negative and Gram-positive bacterial cell walls, showing the location of peptidoglycan (PG) alongside other components in the respective cell walls. Basic concept (with some changes) has been drawn from MartÃ-nez-Carmona, Gun’ko and Vallet-RegÃ, 2018 [115]. Figure 9 has been drawn using ChemDraw software.

The thin layered peptidoglycan of Gram-negative bacteria is sandwiched between the outer membrane (OM) and inner membrane (IM), compared to the multi-layered thick peptidoglycan of Gram-positive bacteria which is located exterior to the cytoplasmic membrane and is exposed to the extracellular environment [114]. Figure 9 is a depiction of Gram-negative and Gram-positive bacterial cell wall types, showing the location of peptidoglycan layers.

Peptidoglycan of the bacterial cell wall serves as a target for many antibiotics [113]. It also serves as a target for antimicrobial amino acids. The incorporation of exogenous d-amino acids into bacterial PG during its biosynthesis disrupts the natural sequence of amino acids in PG and hence produce bactericidal effect [104]. Exogenous amino acids such as d-methionine, d-tryptophan, and d-phenylalanine, when incorporated into bacterial PG, replace l-alanine at position 1 and d-alanine at position 4 and 5 at a terminal position, results in bacterial killing in planktonic or suspended form [116]. Amino acid glycine when provided exogenously, replaces l-alanine at position 1 and d-alanine at positions 4 and 5 in PG, disrupting the natural sequence in it and results in bacterial growth inhibition [117].

Amino acids have been used (in vitro) in combination with antimicrobial drugs and have been shown to have successfully enhanced the efficacy of the drugs against planktonic bacteria [48]. For example, aspartic acid and glutamic acid have been used to improve the efficacy of antibiotic trimethoprim via amino acids-based trimethoprim salts formation [65]. The resultant salts have better solubilities, increased absorption, and hence more effectiveness even at lower concentrations [68].

Different free amino acids and peptides have been reported to exhibit antibacterial activity i.e., nisin, a polycyclic peptide consisted of different amino acids such as alanine, valine, serine lysine, etc., have been shown to have antimicrobial activity against both Gram-positive and Gram-negative bacteria [101,118,119].

Apart from the aforementioned antimicrobial roles of amino acids in planktonic bacterial control, d-forms of different amino acids have been reported to have antibiofilm activity as well, as discussed in the following section.

## 6. d-Amino Acids as Antibiofilm Agents, Adjuvants, and Potential Drugs Excipients

**Figure 10:**
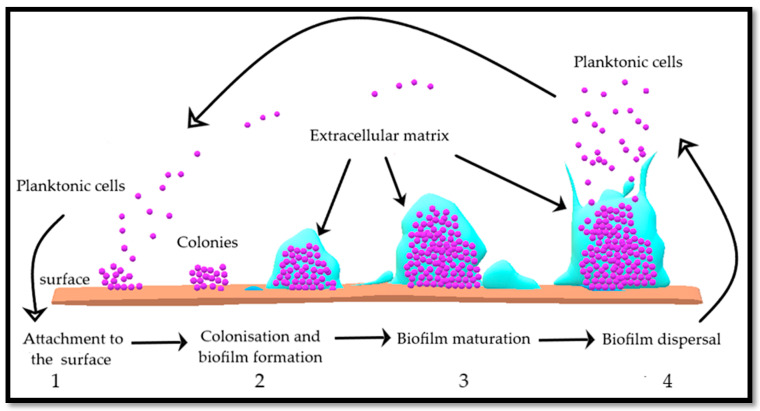
The different stages of microbial biofilm formation are as follows 1) attachment of cells to the surface (host or inanimate surfaces), 2) colonisation and biofilm formation, 3) biofilm maturation and 4) biofilm dispersal. Basic concept has been adopted from Di Luca, Maccari and Nifos, 2014 [124]. Figure 10 has been drawn using Paint 3D app.

Free-living microbial cells attach to the host surface, initiate colonisation and secrete an extracellular polymeric substance called biofilm, composed of polysaccharides, proteins, and DNA (extracellular DNA) [72,120,121]. Biofilm makes it difficult to treat microbial infections as it protects the attached cells residing inside it and thereby offer resistance against antimicrobial drugs [122]. Once a biofilm reaches its maturity, it is dispersed and the microbial cells are released into the surrounding environment, ready to recolonise the host surface and repeat the process of biofilm formation [123], as illustrated in Figure 10.

Prevention and dispersal of microbial biofilm are of utmost importance in treating microbial infections and controlling antimicrobial resistance. Certain enzymes, called racemases facilitate the conversion of l-amino acids into their corresponding d-enantiomers through the process of racemisation [24,125]. These d-enantiomers or d-amino acids have been reported to have the ability to inhibit and disperse microbial biofilms [66]. A mixture of four amino acids, d-leucine, d-tryptophan, d-methionine, and d-tyrosine was able to disperse the microbial biofilm in *Bacillus subtilis* [63]. d-amino acids work via the mechanism of incorporation into the PG of the bacterial cell wall and disrupt the natural sequence of amino acids by replacing d-alanine in PG [103]. This mechanism leads to the release of amyloid fibers from bacterial PG and the dispersal of biofilm [63]. Amyloid fibers are protein fibers that are embedded in bacterial PG and biofilm at opposite ends [126].

d-amino acids were also found to have prevented biofilm formation in *Staphylococcus aureus* and *Streptococcus mutans* [127,128]. Microbial cells are connected to the biofilm via cellulose fibres, embedded into microbial peptidoglycan [129]. The incorporation of d-amino acids into peptidoglycan (during its biosynthesis) disrupts the sequence of existing amino acids and the microfibres-microbial cell linkage breaks up, hence dispersing the biofilm and releasing the sessile microbial cells [63,103].

Amino acids may have potential therapeutic applications as antibiofilm agents and drug excipients as the available literature mentions numerous research studies on the role of amino acids as potential antibiofilm agents and drug excipients. One such study found that the fungicidal and antibiofilm activity of antifungal drug amphotericin B was enhanced when combined (physically) with amino acid lysine, through a possible lysine-mediated generation of endogenous reactive oxygen species (ROS) [130]. The antibacterial and antibiofilm activity of known drugs such as clindamycin, cefazolin, oxacillin, rifampin, and vancomycin (for Gram-positive) and amikacin, colistin, ciprofloxacin, imipenem, and ceftazidime (for Gram-negative) has been reported to have enhanced when combined (physically) with d-amino acids [51].

Amino acids have also been used as adjuvants to enhance the efficacy of inactivated pdm H1N1 vaccine used in the treatment of viral influenza by promoting the cellular uptake of pdm H1N1 and activation of macrophages in the host environment [67,131]. Similarly, the known biocide, hydroxymethyl phosphonium sulphate (THPS) exhibited an enhanced biocidal activity when physically mixed with a mixture of d-tyrosine, d-leucin, d-tryptophan, and d-methionine, compared to the biocidal activity of the THPS alone [132]. One of the possible mechanisms of action for such amino acids-drugs combination is that d-amino acids disperse the microbial biofilm to release and expose the sessile cells (which are now in the planktonic state), thereby making it easier for the drug to reach and eliminate the microbial cells more effectively [133].

A second probable mechanism is the incorporation of D-amino acids into the microbial peptidoglycan that disrupts the sequence of existing amino acids and replace the terminal d-alanine (at fifth position) in the peptidoglycan, enhancing its sensitivity and susceptibility towards antimicrobial drugs [134]. Amino acids have also been used as formulation protectants in freeze-drying [135]. The development of vaccine formulation traditionally involves the use of sugars as cryoprotectants in freeze-drying [136]. However, recent work has reported the use of amino acids as cryoprotectants in freeze-drying, offering similar advantages with improved immune response [137].

Freeze-drying or the lyophilisation technique is employed in the formulation and development of vaccines, mainly to stabilise the vaccines against hydrolysis and physical degradation during storage [138]. However, this technique itself can damage the vaccine during lyophilisation/freeze-drying [139]. To avoid any degradation or damage to the vaccine, amino acids have been used as cryoprotectants. Amino acids being charged molecules, form a protective layer on the surface of liposome by their electrostatic interaction with phosphate head groups of the liposomal lipid molecules, thereby protect the formulation against ice-crystal damage [137].

Despite a variety of pharmaceutical and therapeutic applications, the concentration of amino acids should be carefully optimised in pharmaceutical formulations to avoid any undesired effects. For instance, an overconsumption of amino acids can lead to different undesired consequences such as depressed growth due to antagonism and even toxicity due to high plasma concentrations of the amino acids taken in excess [140].

## 7. Amino Acids as Solubility Enhancing Agents

Solubility is a phenomenon exhibited by any solid, liquid, or gaseous chemical substance with reference to its maximum amount dissolved in a given amount of solvent (solid, liquid, or gas phase) at a specific temperature to form a solution of uniform composition [141,142]. The solubility of chemical substances plays an important role in different scientific fields such as chemistry, biological science, food science, and pharmaceutical science [143].

As the discovery of new drugs continues, about 90% of these potential new drugs have been reported to have poor water-solubility [144]. Poorly water-soluble drugs have problematic absorption and low-bioavailability [142]. Different strategies such as nanotechnology, co-crystals, adsorption enhancers, and salt formation have been adopted to overcome the issue of poor solubility of drugs in aqueous media [145,146].

Amino acids have been reported to have enhanced the solubility of drugs through salt formation technique i.e., solubility of Indomethacin (a nonsteroidal anti-inflammatory drug) was enhanced through salt formation by using basic amino acids l-arginine and l-lysine as counterions [147,148]. ElShaer et al., [65] used acidic amino acids, aspartic acid, and glutamic acid as counterions to form amino acid-trimethoprim salts, enhancing water solubility of trimethoprim by 280 folds.

Similarly, the water solubility of insulin (an antidiabetic drug) and ciprofloxacin was improved by salt formation with cationic and anionic amino acids, respectively [68,149,150]. The increase in solubility of antimicrobial drugs results in improved bioavailability, better absorption and enhanced efficacy of the antimicrobial drugs i.e., the counterion-based salts of antimicrobial drugs are more effective at lower concentrations compared to their free forms [65,144].

Drugs solubility improvement by salt formation with acidic or basic salting agents as counterions, is regulated by parameters such as the acid-base dissociation constant (pKa), pH, and isoelectronic point. The acidity or basicity of a substance is expressed by its pKa value, the acid-base dissociation constant of that substance [151]. The pKa scale provides information regarding the ionizability of a chemical substance and also holds a significant pharmaceutical and chemical importance at an industrial level [152]. The pKa value and acid strength are inversely related i.e., strong acids have low pKa values and vice versa [153]. Moreover, salts that are formed from acidic drugs require counterions with a pKa < pKa of the drugs and salts that are formed from basic drugs require counterions with a pKa > pKa of the drugs [154].

**Figure 11:**
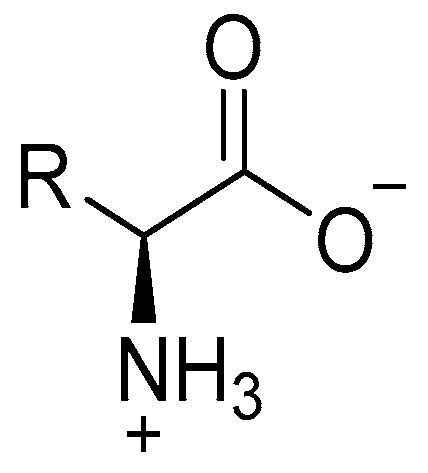
Zwitterionic structure of amino acids.

Amino acids generally exhibit zwitterionic character in aqueous media, therefore, their acidic or basic characters can also be determined by measuring their isoelectronic point (pI). The isoelectronic point, pI of a substance is its pH at which it carries no charge at all i.e., the net charge on substance is equal to zero [155]. Zwitterions represent those compounds that possess both acidic and basic properties, with positive and negative charged species within the same molecule (dipolar) as shown in Figure 11, but the molecule as whole stays neutral [156,157].

An amino acid with a pI less than seven (pI < 7) will exhibit acidic character and an amino acid with a pI greater than seven (pI > 7) will exhibit a basic character [158]. The pKa and pI values for different amino acids are listed in Table 2.

Table 2 shows that the pKa values for α-carboxyl group fall in the range of 1.82–2.21 (acidic), while for α-amino group, the pKa values range from 8.95 to 9.67 (basic). The side chain pKa values of 3.65 for aspartic acid and 4.25 for glutamic acid are representative of their acidic behavior. Similarly, arginine and lysine side chains have higher pKa values i.e., 12.48 and 10.53 respectively, are more basic compared to Histidine with a side chain having a pKa value of 6.

Acidic amino acids i.e., aspartic acid and glutamic acid, have low (2.77 and 3.22) isoelectronic points (pI), compared to basic amino acids such as arginine with a pI value of 10.76, lysine 9.74 and histidine with a value of 7.95. Neutral amino acids have their pI ranging from 5.6 to 5.68.

The pKa and pI parameters are important in amino acid separation, drug solubility determination, predicting permeability of drugs across the microbial cell envelopes and therefore helpful in designing new antimicrobial drugs [64,160,161].

The side chain of an amino acid also determines if a particular amino acid is hydrophilic or hydrophobic, and whether an amino acid will exist as a charged or neutral molecule [162]. Hydrophilic amino acids are water-soluble and tend to exist as positively or negatively charged species due the presence of ionized or polar side chain in their molecules, while the hydrophobic amino acids do not have such side chains and are therefore either insoluble or barely soluble in water [163]. Hydrophobic and hydrophilic amino acids have been listed in Table 3.

The addition of acidic counterions to a poorly soluble basic drug solution usually lowers the pH of the drug solution with salt formation and thereby improves the solubility of the drug, whereas the addition of basic counterions to poorly soluble acidic drugs increase the solution pH and improve drug solubility [65,152,164]. Drugs usually have better solubilities at a pH that is either above or below their pI i.e., at a pH = pI the total charge on the individual solute particles (both counterion and the drug molecules) = zero, a point at which the drug precipitates out in its free from [165,166].

Selection of a suitable amino acid as a counterion for a poorly soluble drug involves a thorough assessment of certain factors such as screening different amino acids for concentration optimisation and selection of oppositely charged amino acids [68,148]. Concentration of amino acid as a counterion affects drug solubility as pH of the drug solution varies with varying amino acid concentration. For instance, amino acids arginine and lysin solutions having concentrations > 100 µg/mL, were able to change the pH of insulin in Hank’s balanced salt solution (HBSS) by almost 2 units i.e., from 7.4 to 9.2, improving insulin’s solubility [149].

Similarly, the overall charge of an amino acid has to be assessed to determine if it can be a suitable counterion for a particular acidic or basic ionisable drug. Amino acids that have overall positive or negative charges are all of hydrophilic nature, with an exception of hydrophobic arginine [162]. Amino acid-based salt formation of a drug requires the selection of appositely charged amino acids to balance the total charge on the resultant salt [167]. These appositely charged counterions interact with acidic or basic ionised drugs in their solutions via strong electrostatic forces of attraction to form stable and neutral salts [154]. The resultant ionic salts have better solubility, improved absorption, and hence enhanced efficacy [65,168].

Amino acids as salt formers also impact the membrane permeability of the resultant salts and thereby affect their overall efficacy. Salt formation often increases the stability and alters lipophilicity of the drug and hence eases the drug permeation across the lipophilic cellular barriers [169]. However, amino acids can be used to improve membrane permeability of drugs by increasing their hydrophilicity via salt formation. For example, one such study has reported aspartic acid to have increased the membrane permeability of antimicrobial drug ciprofloxacin, by enhancing its water solubility through hydrophilic salt formation [68].

Apart from their aforementioned applications, d-forms of amino acids can also be used as sweeteners for taste masking. Since l-amino acids are usually bitter or less sweet compared to their respective d-forms, their racemisation into d-amino acids results in the formation of sweet amino acids or in converting less sweet amino acids into even sweeter ones [170].

## 8. Expert Opinion

Amino acids are naturally occurring substances which play a key role in all living organisms. There are numerous phenotypic studies evaluating the effect of amino acids on growth, metabolism, and control of microorganisms. However, there is a lack of data on the exact mechanism of action of amino acids in various scenarios such as biofilm disruption, synergy with antibiotics, and microbial regulation.

Amino acids present an interesting biological paradox with huge potential in the process of drug development. On one hand, they are required for growth and metabolism in microorganisms, and on the other hand, careful optimisation of composition and concentration can produce antibiofilm and antibacterial effects. The above attribute allows them to be used as drug adjuvants/excipients which ultimately improves the efficacy of drugs. This not only results in better clinical outcomes but also provides a tool to fight antimicrobial resistance which has become a global threat. Improvement in efficacy is achieved by increasing the solubility of poorly soluble drugs and ultimately increasing their bioavailability. As amino acids, when used as excipients/adjuvants, are non-toxic to humans, the development of resulting product/formulation would incur lower costs when compared to developing a new active pharmaceutical ingredient/antibiotic. In addition, understanding the mechanism of amino acid-mediated processes such as disruption of microbial biofilms and their role in enhancing antibiotic activity will enable the development of simple, practical, and cost-effective strategies to reduce AMR. By disrupting biofilms, amino acids will allow increased penetration of antibiotics into the deeper layers of the biofilm, thereby lowering the concentration of the antibiotic required to treat biofilm-related infections. This will result in broadening the use of existing antibiotics to which microorganisms have become resistant thus aiding to repurpose existing antibiotics for a wide range of microbial infections. Impact of such combinations in vivo will have to be established to determine the applicability and safety of amino acid-drug combinations. One of the challenges in this field revealed by reviewing existing literature on the dispersal activity of d-amino acids on biofilms of *Bacillus subtilis, Staphylococcus aureus, Streptococcus mutans,* and *Pseudomonas aeruginosa* is that microorganisms vary in their response to a given amino acid. Therefore, universal mechanism by which amino acids disrupt/disperse biofilms is implausible.

Amino acids have generated considerable interest as biofilm disrupting agents and this strategy has applications in treating biofilm-related infections such as those caused in cystic fibrosis patients and medical device-related infections. The impact of amino acids on biofilms can also be exploited in the food industry and the environment where biofilms are an issue causing spoilage and leading to an increased threat of infection. Thus, amino acids are versatile biomolecules that have a wide range of applications in the pharmaceutical, food, and the industrial environment.

## Figures and Tables

**Table 1:** Precursors involved in microbial biosynthesis of amino acids. Some of the amino acids mentioned in Table 3, do not involve any intermediates and their formation involves their respective precursors only i.e., serine, tryptophan, phenylalanine, valine, etc., while others, such as tyrosine and isoleucine are formed through their respective intermediates. Some amino acids also serve as intermediates in the formation of other amino acids i.e., threonine and phenylalanine serve as intermediates for isoleucine and tyrosine, respectively.

Precursors	Intermediates	Amino Acids
α-ketoglutarate	Glutamate	Glutamine
		Proline
		Arginine
3-phosphoglycerate	Serine	Glycine
		Cysteine
Oxaloacetate	Aspartate	Asparagine
		Methionine
		Isoleucine
		Lysine
Phosphoenolpyruvate + Erythrose 4-phosphate	----------------------	Tyrosine
		Phenylalanine
		Tryptophan
Pyruvate	-----------------------	Alanine
		Valine
		Leucine
Ribose 5-phosphate	------------------------	Histidine

**Table 2:** Enlists all 20 proteogenic amino acids. It highlights acidic, basic and neutral amino acids with their pKa and pI values. Data collected from Haynes, William M., 2014 [159].

Name	Acidic	Basic	Neutral	pKa1 (α-Carboxyl Group)	PKa2 (α-Amino Group)	pKa3 (Side Chain)	pI (Isoelectronic Point)
Alanine			**√**	2.34	9.69		6.00
Arginine		**√**		2.17	9.04	12.48	10.76
Asparagine			**√**	2.02	8.80		5.41
Aspartic acid	**√**			1.88	9.60	3.65	2.77
Cysteine			**√**	1.96	10.28	8.18	5.07
Glutamic acid	**√**			2.19	9.67	4.25	3.22
Glutamine			**√**	2.17	9.13		5.65
Glycine			**√**	2.34	9.60		5.97
Histidine		**√**		1.82	9.17	6.00	7.95
Isoleucine			**√**	2.36	9.60		6.02
Leucine			**√**	2.36	9.60		5.98
Lysine		**√**		2.18	8.95	10.53	9.74
Methionine			**√**	2.28	9.21		5.74
Phenylalanine			**√**	1.83	9.13		5.48
Proline			**√**	1.99	10.60		6.30
Serine			**√**	2.21	9.15		5.68
Threonine			**√**	2.09	9.10		5.60
Tryptophan			**√**	2.83	9.39		5.89
Tyrosine			**√**	2.20	9.11	10.07	5.66
Valine			**√**	2.32	9.62		5.96

**Table 3:** List of different hydrophilic and hydrophobic amino acids with corresponding logP values. The lipophilicity of these amino acids, expressed as their logP, ranges from −4.3 to −1.38, where asparagine with logP value of −4.3 being the least lipophilic and phenylalanine with a logP of −1.38 being the most lipophilic or hydrophobic amino acid. It also shows that hydrophilic amino acids are either charged, uncharged or polar molecules, while hydrophobic amino acids are mostly uncharged and nonpolar.

Hydrophilic Amino Acids	Charged (+/−/Uncharged)	logP	Hydrophobic Amino Acids	Charged (+/−/Uncharged)	logP
**Asparagine**	Uncharged	−4.3	**Arginine**	+	−4.2
**Aspartic acid**	−	−3.89	**Cysteine**	Uncharged	−2.49
**Glutamine**	Uncharged	−3.64	**Isoleucine**	Uncharged	−1.7
**Glutamic acid**	−	−3.69	**Glycine**	Uncharged	−3.4
**Histidine**	+	−3.32	**Phenylalanine**	Uncharged	−1.38
**Lysine**	+	−3.05	**Serine**	Uncharged	−3.07
**Threonine**	Uncharged	−3.5	**Leucine**	Uncharged	−1.6
**Tyrosine**	Uncharged	−2.4	**Valine**	Uncharged	−2.26
